# Genetic counseling and genetic testing for pathogenic germline mutations among high-risk patients previously diagnosed with breast cancer: a traceback approach

**DOI:** 10.1038/s41598-024-63300-8

**Published:** 2024-06-04

**Authors:** Hikmat Abdel-Razeq, Faris Tamimi, Sereen Iweir, Baha Sharaf, Sarah Abdel-Razeq, Osama Salama, Sarah Edaily, Hira Bani Hani, Khansa Azzam, Haneen Abaza

**Affiliations:** 1 Department of Internal Medicine, King Hussein Cancer Center, 202 Queen Rania Al Abdullah Street, P.O. Box: 1269, Amman, 11941 Jordan; 2 School of Medicine, The University of Jordan, Amman, Jordan; 3 CRDF Global, Global Health Mission Area, Amman, Jordan; 4 Office of Scientific Affairs and Research, King Hussein Cancer Center, Amman, Jordan

**Keywords:** Breast cancer, Pathogenic germline mutation, BRCA, Traceback, Genetic counseling, Cancer genetics, Breast cancer

## Abstract

Genetic counseling and testing are more accessible than ever due to reduced costs, expanding indications and public awareness. Nonetheless, many patients missed the opportunity of genetic counseling and testing due to barriers that existed at that time of their cancer diagnoses. Given the identified implications of pathogenic mutations on patients’ treatment and familial outcomes, an opportunity exists to utilize a ‘traceback’ approach to retrospectively examine their genetic makeup and provide consequent insights to their disease and treatment. In this study, we identified living patients diagnosed with breast cancer (BC) between July 2007 and January 2022 who would have been eligible for testing, but not tested. Overall, 422 patients met the eligibility criteria, 282 were reached and invited to participate, and germline testing was performed for 238, accounting for 84.4% of those invited. The median age (range) was 39.5 (24–64) years at BC diagnosis and 49 (31–75) years at the date of testing. Genetic testing revealed that 25 (10.5%) patients had pathogenic/likely pathogenic (P/LP) variants; mostly in *BRCA2* and *BRCA1*. We concluded that long overdue genetic referral through a traceback approach is feasible and effective to diagnose P/LP variants in patients with history of BC who had missed the opportunity of genetic testing, with potential clinical implications for patients and their relatives.

## Introduction

Breast cancer (BC) is the most reported cancer in the Kingdom of Jordan, and represents almost 20% of all reported cancers in the country. It also represents the second most common cancer-related death, after lung cancer^1^. Generally, it is estimated that around 5–10% of all BC cases are hereditary^2,3^; i.e., are attributed to pathogenic germline variants in cancer predisposing genes^2,4^. Such rate can be higher among younger patients and those with significant family history of breast or other cancers^3,5^. In fact, 25% of BC patients have been reported to have a family history of the disease, and having a first-degree relative with the disease is associated with a 1.7–fourfold increased risk of developing it^6,7^. *BRCA1* and *BRCA2* mutations are the most common pathogenic variants (PVs) associated with hereditary BC^8^. Other genes that also are associated with hereditary BC include the high-penetrance genes *TP53, CDH1, PTEN, PALB2, and STK11,* and low/moderate-penetrance genes *ATM, CHEK2, BRIP1*, and *RAD51C*, and *RAD51D*^9–11^. The cumulative incidence rate of BC related to the high-penetrance genes can be as high as 80%, and yet, significant proportion of “familial” clustering of breast cancer remains unexplained^12,13^.

In this regard, genetic testing has become a revolutionary medical practice for BC patients due to its therapeutic implications on systemic treatment, surveillance^14,15^ and surgical decisions^16,17^. These include recommending annual mammograms and MRI screenings of the remaining breast tissue for BRCA1/2 mutation carriers, or risk-reducing surgeries to prevent contralateral BC in *BRCA1/2* PV-carriers^8,18^, use of mastectomy rather than lumpectomy with radiotherapy for BC patients with PVs in *TP53*^19^, and utilizing risk-reducing salpingo-oophorectomy for *BRCA1*/2, *BRIP1*, and *RAD51 C/D-* carrying patients as a preventive approach of subsequent ovarian cancer development, due to its increased association with these PVs^8,20,21^.

Despite the important implications of targeted therapy and surgical interventions, genetic testing and referrals remain underutilized for BC patients^22,23^. A recent study has shown that in the United States, 53% of breast cancer patients who are at high risk for genetic mutations underwent genetic testing, with only 25% of BC survivors reporting undergoing genetic testing at diagnosis^22,24^. The reported reasons for inadequate testing include lack of physician recommendations^24^, reduced awareness among physicians, inadequate time to fully assess family history, and inaccessibility of genetic counseling and testing due to financial restrains^25–27^. Such issues may be even more pronounced in health care systems of developing countries, where genetic testing services are not well established despite refinements in genome sequencing technology and the development of affordable multigene panels for clinical genetic testing^28–32^.

For patients of our facility, genetic counseling and testing for eligible patients have become routinely practiced since 2017. However, it is not offered in other public health care facilities in the country. This presented a potentially life-saving opportunity for our patients through a “traceback” approach, which is a strategy, through the retrospective identification, aimed at identifying BC patients and survivors who were eligible for genetic testing but had not been previously tested. This method involves offering genetic testing to these individuals, identifying probands, tracing their family members, and subsequently offering them genetic counseling and testing^33–35^. The emerging data from our country shows a high rate of PVs/LPVs in our population^36,37^. A higher prevalence of PVs could reflect the younger median age of BC diagnosis, which is 51 years, 10 years younger than the median age of diagnosis in Western countries^38^. Utilizing a traceback approach may provide further insights on the genetic makeup of our unique patient population, in addition to helping identify relatives with PVs who may benefit from cascade testing, and providing BC patients and survivors with certain PVs/LPVs with life-altering risk management treatment options.

## Materials and methods

### Participant identification

The study team identified living BC patients who had been eligible for genetic testing at the time of diagnosis, using our hospital-based cancer registry which compiles records of all patients diagnosed since July 2007. Patient eligibility was based on our institution’s latest guidelines for genetic testing, which conform to the National Comprehensive Cancer Network guidelines for Genetic/Familial High-Risk Assessment Version 2.2021^39^. Accordingly, eligible patients included patients diagnosed with BC at an age of 45 years or younger, patients who were diagnosed with triple-negative BC at 60 years-old or younger, and BC patients diagnosed at age 50 or younger with a first-blood relative with history of BC, between July 2007 and January 2022. We excluded any patient who underwent genetic testing or who had been previously offered genetic counseling and testing.

### Participant recruitment

Eligible patients were contacted by one of the study team members using the contact information available in their electronic medical records, explained the objectives of genetic counseling, obtained verbal agreement for enrollment in a counseling session and testing, and scheduled an in-person visit at our institution's Genetic Counseling Clinic. A 45-min in-person interview was arranged at the clinic as a single visit for each patient, during which a genetic counselor would explain the traceback approach, answer all questions, provide the patient with genetic counseling, conduct a family pedigree, and obtain their written informed consent to have a blood sample drawn for genetic testing.

### Genetic testing methodology

Genetic testing was performed utilizing a peripheral blood sample, DNA was extracted, and testing was performed using a next-generation sequencing (NGS) panel at a reference laboratory. A multi-gene panel was utilized which was comprised of 20 genes: *ATM, BARD1, BRCA1, BRCA2, BRIP1, CDH1, CHEK2, EPCAM, MLH1, MSH2, MSH6, NBN, NF1, PALB2, PMS2, PTEN, RAD51C, RAD51D, STK11* and *TP53*. The genetic testing and sequencing report followed the American College of Medical Genetics and Genomics (ACMG) classification guidance for pathogenic (P), likely pathogenic (LP), variants of uncertain significance (VUS), likely benign, and benign variants^40^.

### Disclosure of genetic test results

Study participants who had a negative genetic testing result were informed by the genetic counselor over the phone. If a patient’s test result revealed P/LPs or VUS, an in-person clinic appointment was arranged to explain the results and their significance and recommend mitigative interventions. Cascade testing for unaffected family members was offered at significantly discounted rates to cover only the cost of specimen shipment and handling.

### Ethics declaration

The study was conducted according to the guidelines of the Declaration of Helsinki, and approved by the Institutional Review Board of King Hussein Cancer Center. All subjects gave their written informed consent for inclusion before they participated in the study.

### Statistical analysis

Clinical and pathological characteristics of patients who underwent genetic testing were tabulated and described by percentages, medians, and ranges. The results of the genetic tests were reported in numbers and percentages. Kruskal–Wallis, Pearson, Wilcoxon testing, and median interquartile ranges were used to compare rates of P/LP and VUS rates among the different study groups. A *p* value < 0.05 was considered statistically significant in all statistical analyses.

## Results

**Figure 1:**
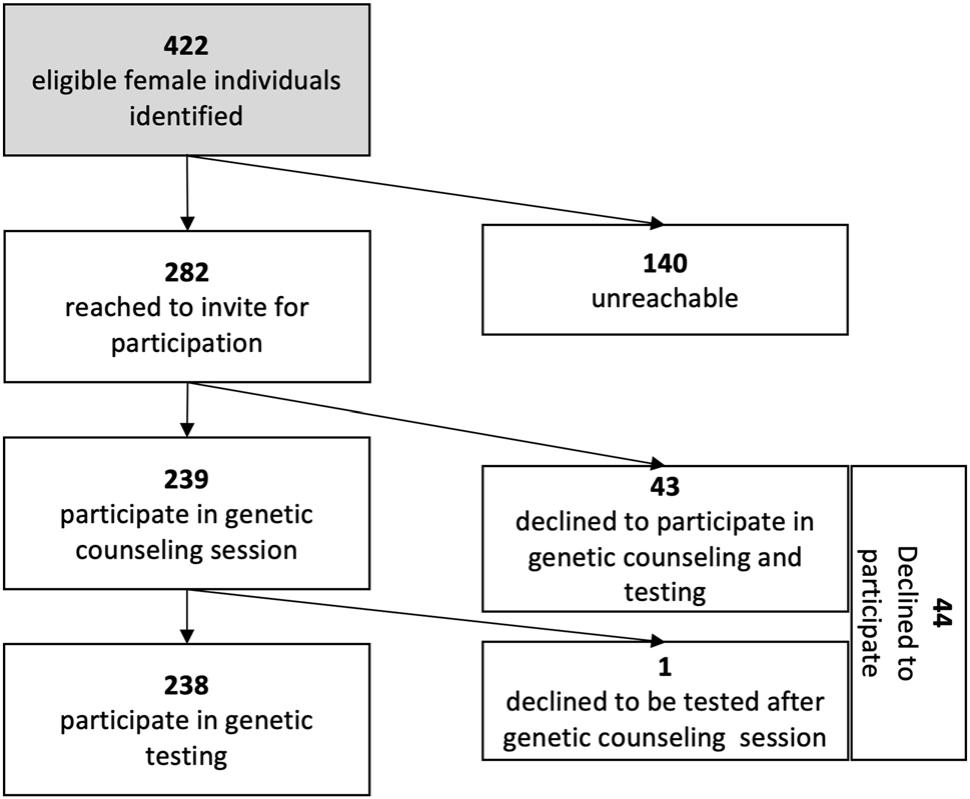
Breakdown of eligible patients as identified in chart review (n = 422) and those who underwent genetic testing (n = 238).

A total of 422 patients with BC met the eligibility criteria through chart review, and all were females. Of which, 140 (33.2%) unreachable or unparticipated, this was due to 58 patients not having updated contact information or being unreachable, 31 who did not follow up at our institution, 27 being out of the country at the time of conducting the study, and 24 missing multiple appointments (Fig. 1).

Of the 282 individuals who could be tested, 44 (15.6%) declined to provide a sample for testing due to: patient disinterest, physical burden, inactive insurance at our institution, social fears, and emotional stress (as reported by 13, 12, 8, 6, and 5 patients, respectively), Table 1.

**Table 1:** Reasons for failing or declining genetic testing among patients eligible for traceback genetic testing.

Reason	Number	Percentage (%)
Failed to be tested (n = 140)		
No updated contact information or unreachable	58	41.4
Follow up at other institutions	31	22.1
Outside the country	27	19.3
Missed multiple appointments	24	17.1
Declined genetic test (n = 44)		
Patient disinterest	13	29.5
Physical burden	12	27.3
Inactive insurance at our institution	8	18.2
Social fears	6	13.6
Emotional stress	5	11.4

Of the 238 individuals participated in genetic testing (Fig. 1), the most common indication for study inclusion was being diagnosed with BC at 45 years or younger, which was the case for the majority of the participants (n = 185, 77.7%), followed by a triple-negative BC diagnosis at the age of 60 or younger for 70 (29.4%) patients, while 44 (18.5%) patients were included due to having one or more first-degree relatives with BC and were 50 years or younger at diagnosis. Sixty-one (25.6%) patients met more than one eligibility criterion.

The median age for the study population was 39.5 (24–64) years at BC diagnosis, and 49 (31–75) years at the time of genetic testing, resulting in a median time of 8 (2–20) years between diagnosis and genetic testing as part of this study. The majority (97.5%, n = 232) of the patients were disease-free at the time of genetic testing.

The chart review of the BC pathology reports for the patients who underwent genetic testing revealed that 225 (94.5%) had invasive ductal carcinoma, and 83 (34.9%) had high-grade pathology at the time of their initial diagnosis. In addition, 147 (61.8%) of the patients had estrogen receptor positive disease, 40 (16.8%) HER2/neu positive, and 73 (30.7%) had triple-negative disease. The characteristics of the patients tested are presented in Table 2.

**Table 2:** Clinical and demographical characteristics of study participants (n = 238).

Age (years)	Median	Range
Age at genetic testing	49	31–75
Age at first diagnosis with breast cancer	39.5	24–64
Time between diagnosis and genetic testing (years)	8	2–20

Receptors	Number	Percentage (%)
Estrogen receptors		
Negative	91	38.2
Positive	147	61.8
Progesterone receptors		
Negative	100	42.0
Positive	138	58.0
HER2/neu		
Negative	184	77.3
Positive	40	16.8
Unknown	14	5.9
Triple-negative	73	30.70
Grade		
High	83	34.9
Intermediate	58	24.4
Low	13	5.5
Unknown	84	35.3
Pathology		
IDC	225	94.5
ILC	4	1.7
Other	9	3.8
Stage at diagnosis		
Non-metastatic	232	97.5
Metastatic	6	2.5
Indications for testing^a^		
Diagnosed at age ≤ 45	185	77.7
Diagnosed at age ≤ 60 and triple-negative disease	70	29.4
≥ One close blood relative with breast cancer at age ≤ 50	44	18.5

*HER2/neu* human epidermal growth factor receptor 2, *IDC* invasive ductal carcinoma, *ILC* invasive lobular carcinoma.

^a^The same patient may have more than one indication.

### Genetic testing results

**Figure 2:**
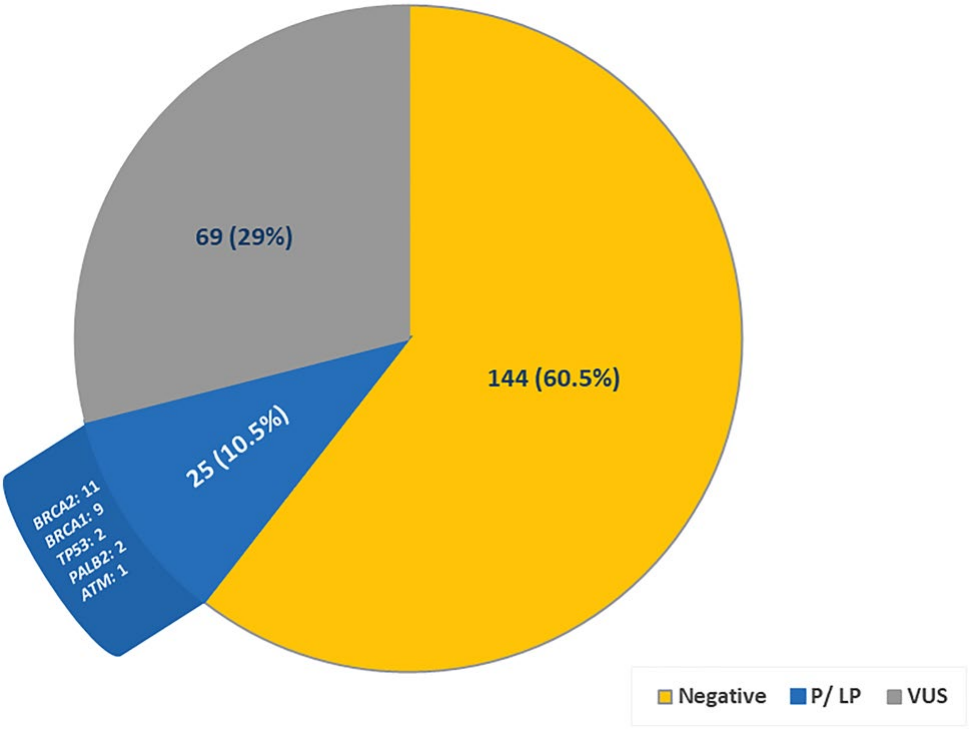
Results of traceback genetic testing for 238 breast cancer patients. *P/LP* pathogenic/likely-pathogenic variants, *VUS* variants of uncertain significance.

P/LP variants were identified in 25 (10.5%) patients, namely, *BRCA2* (n = 11), *BRCA1* (n = 9), *TP53* (n = 2), *PALB2* (n = 2) and one case with *ATM*. On the other hand, 69 (29.0%) patients expressed VUS (Fig. 2). The frequency of P/LP-positive results significantly varied among the different age groups of BC diagnosis. The highest rate (22.7%) was among patients who were younger than 39 at diagnosis (*p* = 0.003), while age at BC diagnosis was statistically nonsignificant for patients who had VUS (*p* = 0.52).

Diagnosis with triple-negative breast cancer was not significantly associated with P/LP nor VUS (*p* = 0.55 and 0.72, respectively). Additionally, while having a record of any family history of cancer among first-, second-, or third-degree relatives was not significantly different among the P/LP and VUS cohorts, patients with two first-degree relatives who had positive BC diagnoses were significantly more likely to present with positive P/LP results (*p* = 0.0002). Statistical analyses results are summarized in Table 3.

**Table 3:** Genetic testing results based on age group and family history (n = 238).

Characteristics	VUS (n = 69)	*P* value	P/LP (n = 25)	*P* value
Age at BC diagnosis				
≤ 39 (n = 44)	11 (25.0%)	0.52	10 (22.7%)	0.003
40–49 (n = 136)	41 (30.1%)		11 (8.1%)	
50–59 (n = 38)	13 (34.2%)		2 (5.3%)	
≥ 60 (n = 20)	4 (20.0%)		2 (10.0%)	
Indication for genetic testing				
Triple negative BC				
Yes (n = 73)	20 (27.4%)	0.72	9 (12.3%)	0.55
No (n = 165)	49 (29.7%)		16 (9.7%)	
Any family history of cancer in 1st, 2nd, or 3rd degree relative				
Yes (n = 175)	51 (29.1%)	0.94	22 (12.6%)	0.084
No (n = 63)	18 (28.6%)		3 (4.8%)	
Two close relatives with BC at any age				
Yes (n = 25)	10 (40.0%)	0.2	8 (32.0%)	0.0002
No (n = 213)	59 (27.7%)		17 (8.0%)	

*BC* breast cancer, *P/LP* pathogenic/likely pathogenic variants, *VUS* variants of uncertain significance.

## Discussion

To our knowledge, this is the first study in the Middle East and North African (MENA) region that evaluated the feasibility and effectiveness of using the traceback approach to diagnose P/LP variants in individuals with a history of BC who had otherwise missed the opportunity of genetic counseling and testing at the time of diagnosis. Despite a median interval of 8 years between the initial cancer diagnosis and genetic testing, the rate of P/LP variants among our patients was 10.5%, comparable to the P/LP rates observed in upfront testing for breast cancer patients deemed eligible at diagnosis. These findings affirm that delayed genetic assessments can still be clinically relevant and effective^36,37^. In addition, the study results highlighted important patterns to patients with P/LPs and VUS and provided an opportunity for further testing and risk reduction strategies for relatives of patients who were otherwise unaware of the highly consequential mutations they may be carrying.

It is worth first noting the relatively young age of our patient cohort, who had a median age of 39.5 years-old at BC diagnosis. This observation aligns with the trend noted among Jordanian women, who are typically diagnosed with BC at a median age of 51 years, a decade earlier than their counterparts in Western nations^38^. Age appeared to have an impact on test result outcomes among our patients, with notable variations in patients with P/LP observed across different age groups. P/LP variants were particularly prevalent among individuals aged 39 years old or younger. This is consistent with previous reports associating younger BC patients with a higher predisposition for hereditary etiology. Reports have estimated that 12% of BC patients who developed the disease at ≤ 40 years were linked with PVs in *BRCA1* or *BRCA2* genes^41–43^, compared to global estimates of 3–4% of all women with BC^18,44,45^. Moreover, an analysis by Daly et al. of 130,151 BC patients using a 25-gene panel revealed that young age (≤ 45 years) at diagnosis was strongly associated with presence of PVs for *BRCA1* (OR 3.95, 95% CI 3.64–4.29), moderate for *BRCA2* (OR 1.98, 95% CI 1.84–2.14), modest associations for *ATM* (OR 1.22, 95% CI 1.08–1.37) and *CHEK2* (OR 1.34, 95% CI 1.21–1.47) genes, and not associated with PVs for *PALB2* (OR 1.12, 95% CI 0.98–1.27)^46^. Future prospective analyses of BC patients from our region could provide further insights to the prevalence of specific P/LPs among early-onset cancer patients.

The vast majority of the study participants (97.5%) presented with non-metastatic disease at diagnosis, and all individuals identified as carriers of P/LP variants were disease-free at the time of genetic testing. These carriers stand to gain from personalized screening, risk-reduction strategies, and family planning. Furthermore, cascade testing is clearly warranted, offering their family members the opportunity of genetic counseling and testing, and potentially earlier detection and prevention of cancer^47^. Most importantly, the identification of P/LP variants among our cohort of patients with history of BC provided an opportunity for significant risk mitigation procedures. Since P/LPs variants in *BRCA1, BRCA2, CHEK2,* or *PALB2* substantially increase the risk of contralateral BC, the patients may benefit from additional aggressive surveillance specifically for contralateral BC with supplemental magnetic resonance imaging (MRI) and mammograms, as per international guidelines^48^. In addition, since P/LPs in *BRCA1/2* have been associated with increased risk of subsequent ovarian cancer in BC survivors, our patients with positive P/LPs in these genes may benefit from prophylactic salpingo-oophorectomy^20^. The findings of this study could also guide the type of risk reduction surgery a patient might undergo, as is the case for BC patients with *TP53* PVs, for which mastectomies are preferred over lumpectomies^19^. On the other hand, VUS were found in 69 (29%) of our subjects. Although the majority of VUS reclassification downgrade variants to benign or likely benign^49,50^, the result is noteworthy as VUS could place an additional burden on individuals and the health care system, by requiring regular reviews for potentially reinterpreted genetic test results.

Furthermore, the characteristics of our patient population addressed the feasibility of identifying eligible individuals for genetic testing based on age and pathology (i.e., triple-negative BC) as per the NCCN guidelines followed in the study design. Indeed, of the 238 identified eligible BC patients, 185 (77.7%) were diagnosed with breast cancer at aged 45 years or younger, and only 70 (29.4%) had triple-negative disease aged 60 years or younger. However, major challenges were observed in identifying eligible patients based on family history alone, as this process usually relies on a patient’s medical records, which tended to be less extensive in the early years of the study period. This was exacerbated by the fact that physicians rarely updated the family history records of patients who had completed active cancer treatment despite potential changes to their relatives’ health status over time. In fact, only 14 (0.6%) of the eligible patients were identified based on family history records alone. This observation presents yet another example to the importance of comprehensive noting and regular review of cancer patients’ family history records^51^.

## Limitations

While this study provides promising insights into the feasibility and efficacy of the traceback approach for genetic testing in BC patients and survivors who were eligible for genetic testing but had not been previously tested, several limitations must be considered; the study only included patients who were still alive and willing to undergo genetic testing, which may introduce a survival bias. Patients who have died since their initial diagnosis or those who were not reachable or declined participation might have different genetic test results, potentially skewing the prevalence and spectrum of P/LP variants observed. The retrospective nature of the study limits the ability to control for variables that might influence the results, such as changes in eligibility criteria for genetic testing over time; this may affect the generalizability of the findings to current patients.

The study lacks long-term follow-up data to evaluate how genetic findings affected patient treatment and family risk management. It also does not detail the identification of at-risk relatives via cascade testing, or the effectiveness of the risk-reduction strategies used. These limitations stem from the study starting before the Early Detection and Prevention Clinic was established at our center in late 2022. We did not report results for patients tested according to the guidelines since the availability of genetic counseling and testing since 2017 to compare with the traceback approach. Additionally, the economic effects of implementing traceback genetic testing were not assessed.

## Conclusions

Tracing back individuals with history of BC who had missed the opportunity to undergo genetic testing to diagnose P/LP variants, is a feasible and effective method that can provide actionable information for both patients and their relatives, and guide personalized treatment decisions and preventative measures, even after long overdue genetic referral. P/LP rates were found to be comparable to the rate of P/LP in upfront testing for eligible breast cancer patients in our country^52^.

## Data Availability

Data generated during and analysed during the current study are available from the corresponding author upon request. All variants reported in this manuscript, are submitted regularly to ClinVar. Our most recent submission has been processed in March 2023. Details are available at: https://www.ncbi.nlm.nih.gov/clinvar/submitters/500031.
